# Investigating host-bacterial interactions among enteric pathogens

**DOI:** 10.1186/s12864-019-6398-2

**Published:** 2019-12-27

**Authors:** Tungadri Bose, K. V. Venkatesh, Sharmila S. Mande

**Affiliations:** 1 Bio-Sciences R&D Division, TCS Innovation Labs, Tata Consultancy Services Limited, Pune, India; 2 Department of Chemical Engineering, Indian Institute of Technology Bombay, Mumbai, India

**Keywords:** Gut pathogens, Host pathogen interactions, Protein interaction networks, Therapeutic targets

## Abstract

**Background:**

In 2017, World Health Organization (WHO) published a catalogue of 12 families of antibiotic-resistant “priority pathogens” that are posing the greatest threats to human health. Six of these dreaded pathogens are known to infect the human gastrointestinal system. In addition to causing gastrointestinal and systemic infections, these pathogens can also affect the composition of other microbes constituting the healthy gut microbiome. Such aberrations in gut microbiome can significantly affect human physiology and immunity. Identifying the virulence mechanisms of these enteric pathogens are likely to help in developing newer therapeutic strategies to counter them.

**Results:**

Using our previously published in silico approach, we have evaluated (and compared) Host-Pathogen Protein-Protein Interaction (HPI) profiles of four groups of enteric pathogens, namely, different species of *Escherichia*, *Shigella*, *Salmonella* and *Vibrio*. Results indicate that in spite of genus/ species specific variations, most enteric pathogens possess a common repertoire of HPIs. This core set of HPIs are probably responsible for the survival of these pathogen in the harsh nutrient-limiting environment within the gut. Certain genus/ species specific HPIs were also observed.

**Conslusions:**

The identified bacterial proteins involved in the core set of HPIs are expected to be helpful in understanding the pathogenesis of these dreaded gut pathogens in greater detail. Possible role of genus/ species specific variations in the HPI profiles in the virulence of these pathogens are also discussed. The obtained results are likely to provide an opportunity for development of novel therapeutic strategies against the most dreaded gut pathogens.

## Background

The human gut harbors more than 100 trillion microbial cells belonging to over a 1000 phylotypes [1, 2]. This microbial community, referred to as the ‘human gut microbiota’, is known to impact human physiology, metabolism, nutrition and immune functions [1, 3]. The human gut (enteric) microbiota is predominated by commensal species belonging to phyla Firmicutes and Bacteroidetes [1, 3]. Although pathogenic bacteria (like *Campylobacter jejuni*, *Salmonella enterica*, *Vibrio cholera*, *Escherichia coli*, and *Bacteroides fragilis*) are also observed in gut microbiota, their abundances are significantly lower in healthy human gut [3]. Interestingly, species like *Helicobacter pylori*, which are otherwise commensal (and are constituents of the healthy gut microbial community), can acquire pathogenic phenotype under certain conditions [3]. The manifestation of virulence in pathogenic bacteria is usually mediated through small molecules (such as bacterial toxins) and cell surface associated and secreted proteins, which in turn interact with the host proteins.

In 2017, World Health Organization (WHO) published a list of pathogens [4] perceived to pose greatest threat to humanity. The report identified growing antimicrobial resistance of these pathogens as the major cause of concern. The associations between the host and various pathogens are governed by several factors which include expression patterns of bacterial gene/ protein as well as the availability of metabolites in the environmental niche that is inhabited by the pathogens. Studying ‘Host-Pathogen Protein-Protein Interactions’ (HPIs) can help in understanding (at least in part) the probable mechanisms of infections adopted by different pathogens [5, 6]. Notably, six of the 12 WHO enlisted pathogens [4] correspond to enteric diseases, thereby highlighting the need to understand the probable biological mechanisms of the enteric pathogens (including their interactions with host) in greater detail.

HPI studies may either focus on an in-depth understanding of a particular aspect of pathogenesis or a host defense mechanism involving one (or a few) protein(s) from the host and pathogen [7–10], or may aim at obtaining a systems level view of the host-pathogen interplay [11–14]. While the former relies primarily on experimental approaches to validate a hypothesis, the latter can be studied using genome-scale bioinformatic approaches. The said bioinformatics approaches typically rely on a template protein-protein interaction (PPI) library for inferring HPIs among the host and a pathogen [5, 6, 14]. HPIs from template PPI libraries can either be inferred using (a) structural similarity [15–19] and/ or by (b) sequence similarity based methods [5, 6, 14, 20, 21]. Thus the choice of tool to study HPI is usually determined by the question(s) to be addressed. The objective of the current study was to perform an in silico comparative analysis of the HPI profile of different gut pathogens. The current investigation therefore used a genome-scale bioinformatics approach. Given the scarcity (and un-uniformity) in the availability of good quality 3-D structures of human proteins as well as of all the studied pathogens, a sequence similarity based methodology was preferred over a structural similarity based approach.

In our earlier published study, we reported that different strains of *E. coli* share a common repertoire of interactions with the host, irrespective of the pathogenic nature [5]. Parasites with similar sites of infection have also been shown to share common features in their host-parasite interaction networks [22]. Since all microbes residing in the human gut share a common environment, we speculated that such similarities in HPI profiles could also exist among most (if not all) bacterial groups residing in human gut. The HPI profile of different enteric pathogens were accessed to check if there were any underlying commonalities in their interactions with the host. Such findings could be utilized for devising broad-spectrum antimicrobial strategies against these pathogens. In this work, we have performed an in silico analysis of HPIs pertaining to different enteric pathogens (namely, different species of *Escherichia*, *Shigella*, *Salmonella* and *Vibrio*) with an objective to decode their pathogenic mechanisms. We could identify a common repertoire of bacterial protein which is possibly involved in the microbes’ adaptation to the niche environment within the gut. In addition, bacterial proteins involved in genus/ strain specific HPIs with the host could also be found. This identified set of bacterial proteins may be used as potential therapeutic targets for narrow-spectrum antimicrobial development. Thus knowledge obtained from this study is likely to help in better understanding of the virulence processes adopted by different enteric pathogens and is likely to provide a road map for future studies in this direction.

## Results

### Host-pathogen interactions (HPIs) involving human cells and enteric pathogens

The number of host-pathogen interactions identified for each of the studied strains is presented in Table 1 (details in Additional file 1). The number of host and bacterial proteins involved in the HPIs was observed to be substantially lower in *V. cholerae* strains as compared to other pathogens. Further, in concordance with their generic phenotypes [23], the number of interactions pertaining to *Shigell*a strains was noted to be similar to those of *E. coli*. However, interaction patterns involving *S. enterica* strains were found to differ from that in *E. coli*. Notably, as compared to the *S. enterica* serovar Typhi species the *S. enteric* serovar Typhimurium species demonstrated a higher number of HPIs with the human. The possible roles of this additional set of HPIs in *S. enteric* serovar Typhimurium infection has been discussed in a later section. In line with an earlier literature [5], a number of host and pathogen proteins were seen to have high degrees of interaction in the corresponding host-pathogen interaction networks (Additional file 2). As was indicated in our earlier study [5], such high degree nodes are usually associated with crucial biological functions. Interestingly, some of these high degree nodes (in the present study) were found to be absent in some of the analyzed HPI networks. For example, amongst the studied enteric pathogens, HPIs involving UDP-sugar hydrolase (UshA) in *S. enterica* serovar Typhi strains was absent. Similarly, HPIs involving acyl-CoA thioesterase I (TesA) was absent in *Shigella flexneri* 301. Also the HPI sub-network involving three high degree bacterial proteins (AbcT2, AbcT3, AbcT5) belonging to the HlyB subfamily of ABC transporter in two studied *V. cholerae* strains (O395 and N16961) were found to be absent in the HPI profiles of all the other studied bacterial strains. It is to be noted that HlyB family of ABC transporter proteins have previously been shown to play vital roles in the secretion of hemolysins which is crucial for invasion of the host’s intestinal villi by *V. cholerae* [24].

**Table 1:** Statistics of the number of observed protein-protein interactions (PPIs) involving human (host) and bacterial (pathogen) proteins, among the studied bacterial strains

Interacting bacterial strain	Total number of interactions	Total number of proteins involved	Number of interacting bacterial proteins	Number of interacting human proteins	Average degree of nodes (proteins)
*Salmonella enterica subsp. enterica* serovar Typhi CT18	582	273	104	169	4.20
*Salmonella enterica* subsp. enterica serovar Typhi Ty2	582	273	104	169	4.20
*Salmonella enterica* Serovar Typhimurium LT2	634	279	109	170	4.48
*Escherichia coli* O157 H7 EC4115	762	283	118	165	5.29
*Escherichia coli* O157 H7 EDL933	773	292	122	170	5.21
*Escherichia coli* O157 H7 Sakai	771	289	120	169	5.25
*Escherichia coli* O157 H7 TW14359	764	286	120	166	5.25
*Escherichia coli* K-12 MG1655	783	291	120	171	5.29
*Shigella dysenteriae*	744	265	102	163	5.52
*Shigella flexneri* 301 (serotype 2a)	703	269	108	161	5.14
*Vibrio cholerae* O1 biovar El Tor N16961	346	154	54	100	4.49
*Vibrio cholerae* O395	357	155	55	100	4.61

In general, the host and the bacterial proteins which demonstrated high degree centrality in the HPI-network were also characterized by high betweenness centralities. However, certain bacterial and human proteins were found to have high betweenness values in spite of their low degree centralities (Additional file 2). The topological architecture of such proteins (nodes) in the HPI-network was indicative of their central role in the infection process [5]. Noticeably, betweenness centralities values of some of the human proteins, such as O-sialoglycoprotein endopeptidase like 1 (OSGEPL1), V-type proton ATPase subunit B (ATP6V1B2) and acid phosphatase 1 (ACP1) were seen to vary between the studied strains (biological implications are discussed later). The above observations are probably indicative of the selective adaptation of different groups of bacterial pathogens to survive and persist inside the host.

In an attempt to understand the probable role of the identified HPIs in the overall infection process for each of the studied organisms, analyses of the KEGG infection pathways with respect to the human proteins involved in the HPIs were performed. The KEGG database [25, 26] contains literature curated information on the human proteins that are affected during bacterial infection processes. Since information pertaining to *V. cholerae* infection process was not available in the database, the analyses were restricted to understanding the virulence processes of *E. coli*, *S. enterica* and *Shigella*. It was observed that for all the studied organisms (Additional file 3: Appendix 1), there was a significant overlap between the human protein set which interacted with the pathogens (as per the HPI analysis) and the human proteins which were involved in the infection process (reported in KEGG). A few genus and species specific differences were also observed. For example, proteins associated to human Toll-like receptor signaling pathway was found to be more intricately associated with the *E. coli* infection process as compared to infection by other pathogens. Further, mechanism of actin rearrangement of human epithelial cells was found to be different in response to *S. flexneri* infection when compared with *S. dysenteriae* infection.

### Common interactions between host and different enteric pathogens

**Fig. 1:**
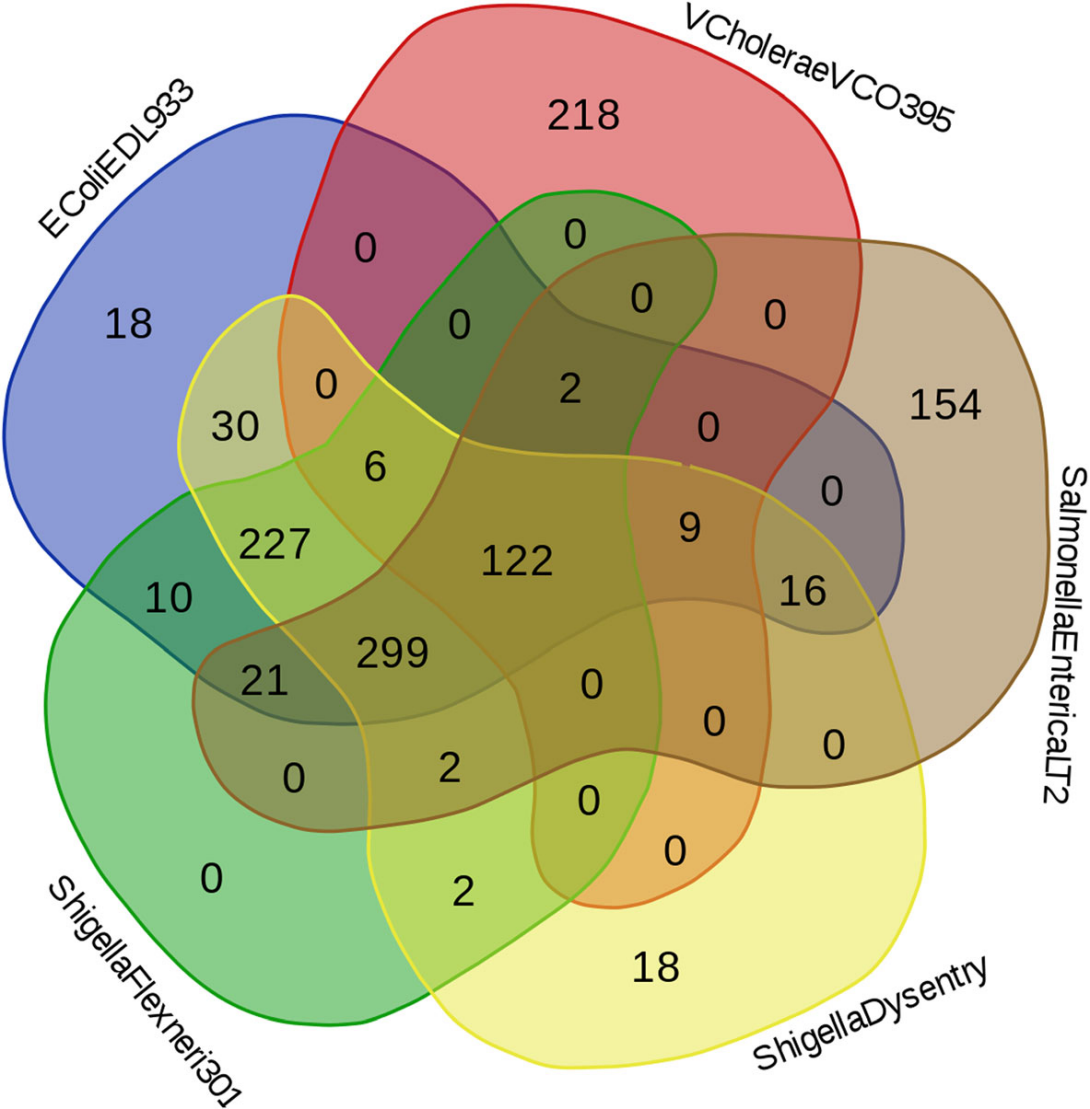
Euler diagram representing the number of HPIs involving human proteins and those from the different studied enteric pathogens. A total of 122 PPIs involving 17 bacterial and 122 human proteins were common to all the studied pathogens

Irrespective of their mode of infection, all enteric pathogens enter the host alimentary system and adapt to the harsh environment inside the human gut. Upon entry inside the host, these pathogens need to cope up with the host’s bile acids, immune defenses as well as a nutrient scarce environment. It was therefore speculated that the enteric pathogens probably adopt similar strategies (including their interactions with host) to deal with the adversities posed by their host. Analysis of the HPI profiles of the studied pathogens revealed a total of 122 PPIs which were common to most of the studied pathogens (Fig. 1). The interactions included bacterial proteins like UDP-sugar hydrolase (UshA), arginine-binding periplasmic protein 1 (ArtI), transferrin binding protein A (TbpA), cytosol aminopeptidase (PepA), cytosol non-specific dipeptidase (PepD), aminopeptidase N (PepN), glutathione reductase (Gor), glutathione synthetase (GshB), ABC transporter periplasmic-binding protein (SapA), gamma-glutamyltransferase (Ggt) etc. (Additional file 4A). Experimental evidences supporting the functional importance of a few of these bacterial proteins and/ or their involved in interactions with the host could be obtained from earlier literatures. For example, significant reduction in CFUs of *S. enterica* serovar Typhimurium in aminopeptidase N (PepN) mutants as compared to wild type was reported in a study which inspected systemic infection in mice models [27]. Further, the bacterial proteins involved in this subset of interactions were found to be enriched in glutathione and sulfur metabolism (Additional file 4A). Given the role of glutathione in innate immunity and inflammation [28], the ability to abrogate glutathione mediated stress is probably important for a pathogen to survive inside the host. Further, sulfur containing compounds are often found to be associated with biological pathways leading to detoxification of reactive oxygen species (ROS) and glutathione [29, 30]. Moreover, in line with the expectations, bacterial superoxide dismutase (SodB) was also found to occur among the core interactors in the HPI networks. The other bacterial proteins in this sub-network were UshA and SapA. While UshA is involved in abrogation of host immune defenses [31–33], SapA has been shown to be play roles in neutralization of antimicrobial peptides (AMPs) [34].

**Fig. 2:**
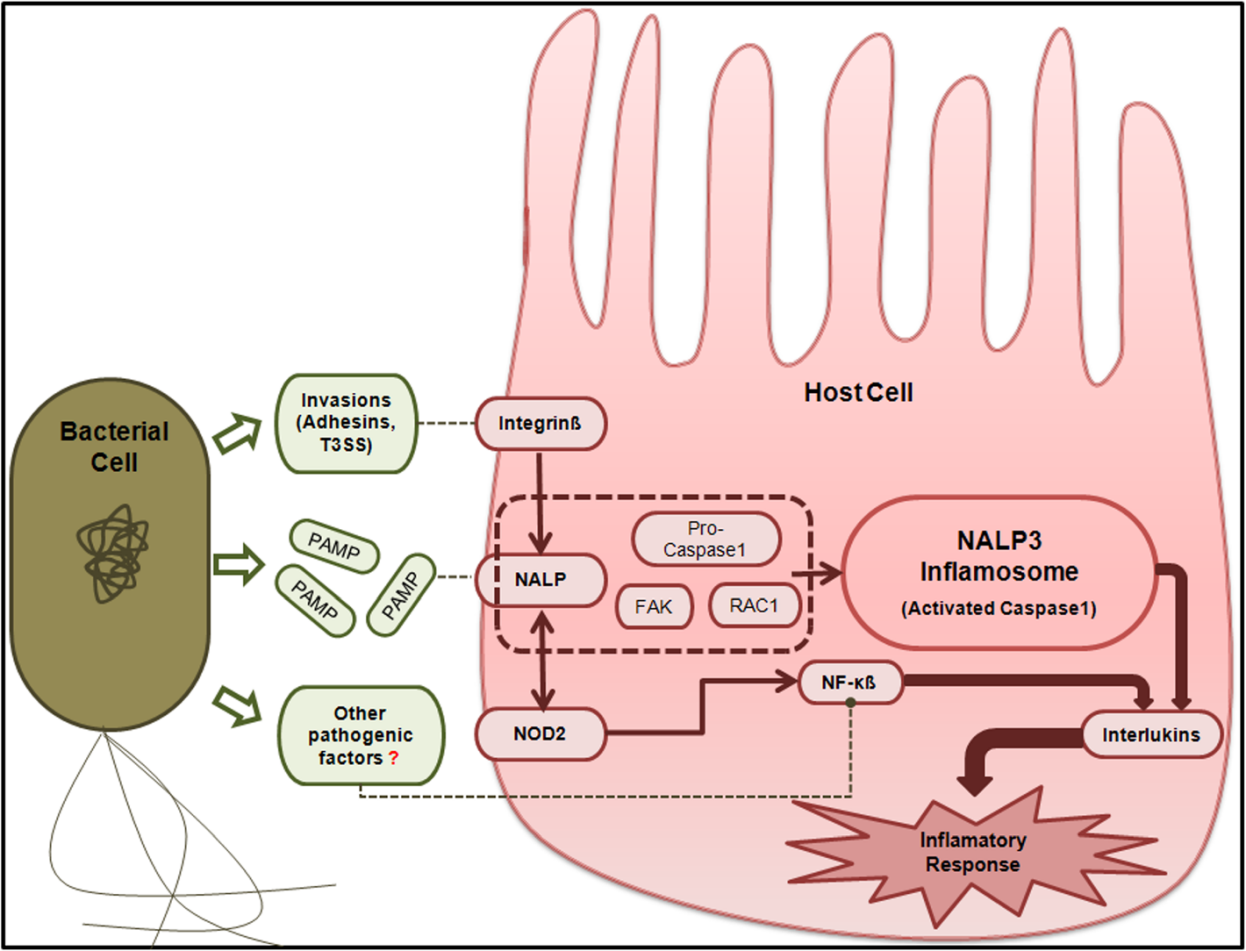
Probable role of the human proteins (and their neighbors), involved in the core set of HPIs with enteric pathogens, in the activation of inflamsomes in intestinal epithelium cells. NALP3 is a pathogen recognition receptor of the NOD-like receptor (NLR) subfamily. It functions by recognizing pathogen-associated molecular patterns (PAMPs). NALP3 together with proteins like PYCARD/ ASC forms a caspase-1 activation complex known as the NALP3 inflamasome. Activation of NALP3 inflamasome further requires the assistance of focal adhesion kinase and rac 1 from the focal adhesion complex signaling pathway. Interaction of host integrinβ with pathogenic factors (like invasions) acts as the first signal for the activation of NALP3 inflamasomes. The second set of signals for the activation of inflamosomes is mediated through the Type III secretion system translocon. Expression of NALP3 inflamasome in turn results in the release of interleukins, especially IL-18. NALP3 also interacts with NOD2 leading to activation of interleukins through a pathway independent of the caspase recruitment domain-containing proteins. Inhibition of NF-κB is a common strategy adopted by enteric pathogens to block the integrin signaling pathway, thereby evading host’s immune and inflammatory responses.

The human proteins involved in this subset of interactions were found to be enriched in ATP driven trans-membrane movement of substances, nucleotide metabolism and cofactor metabolism (Additional file 4B). The average degree centrality of these human proteins was observed to be substantially lower than the rest of the nodes (proteins) in the network. In order to evaluate whether this set of interactions was biologically meaningful or simply an artifact, this subset of proteins (along with their 1st degree neighbors) was plotted on the KEGG infection pathway. The proteins were found to be associated with the mechanism related to activation of inflamsomes in intestinal epithelium cells. This was also elucidated in a few previously published literatures [35–38]. The probable role of these human proteins (which were observed in the core set of HPIs with enteric pathogens and their neighbors) in the mechanism of activation of inflamsomes in intestinal epithelium cells is illustrated in Fig. 2. While the host proteins involved in the HPIs is likely to play key roles in the initiation of the immune responses, the bacterial proteins participating in these HPIs could potentially aid the pathogen in evading host immunity in order to successfully colonize inside the host.

### Variations in interactions (with host) of enteric pathogens belonging to the same genera

**Fig. 3:**
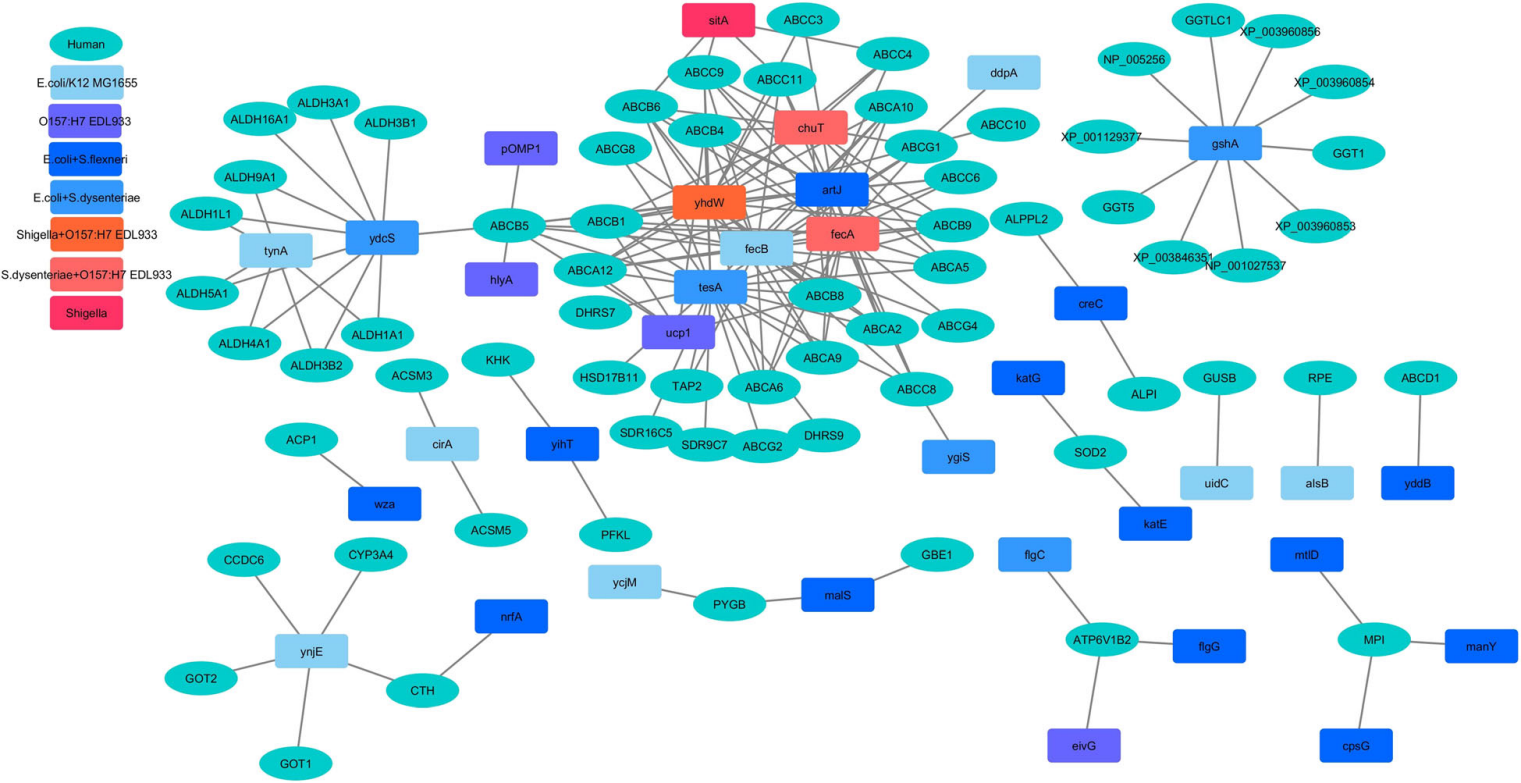
Comparative analysis of the HPI-networks of studied *Escherichia coli* and *Shigella* strains. Depiction of the differences in the HPI network, along with the involved human (host) and bacterial (pathogen) proteins have been shown. 637 interactions that were common to all the studied stains of *Escherichia coli* and *Shigella* have not been shown in this figure

**Fig. 4:**
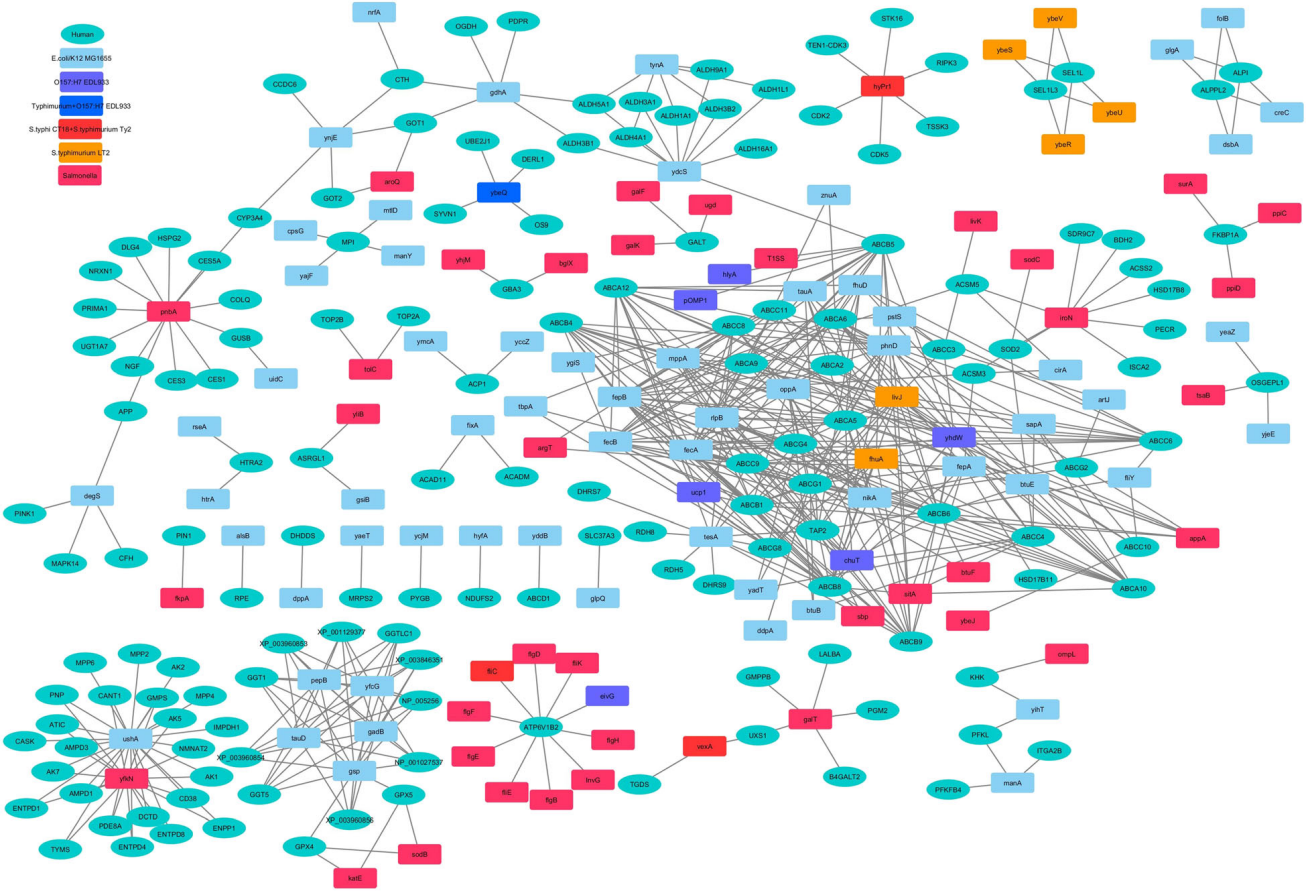
Comparative analysis of the HPI-networks of studied *Escherichia coli* and *Salmonella* strains. Depiction of the differences in the HPI network, along with the involved human (host) and bacterial (pathogen) proteins have been shown. 418 interactions that were common to all the studied stains of *Escherichia coli* and *Salmonella* have not been shown in this figure

**Fig. 5:**
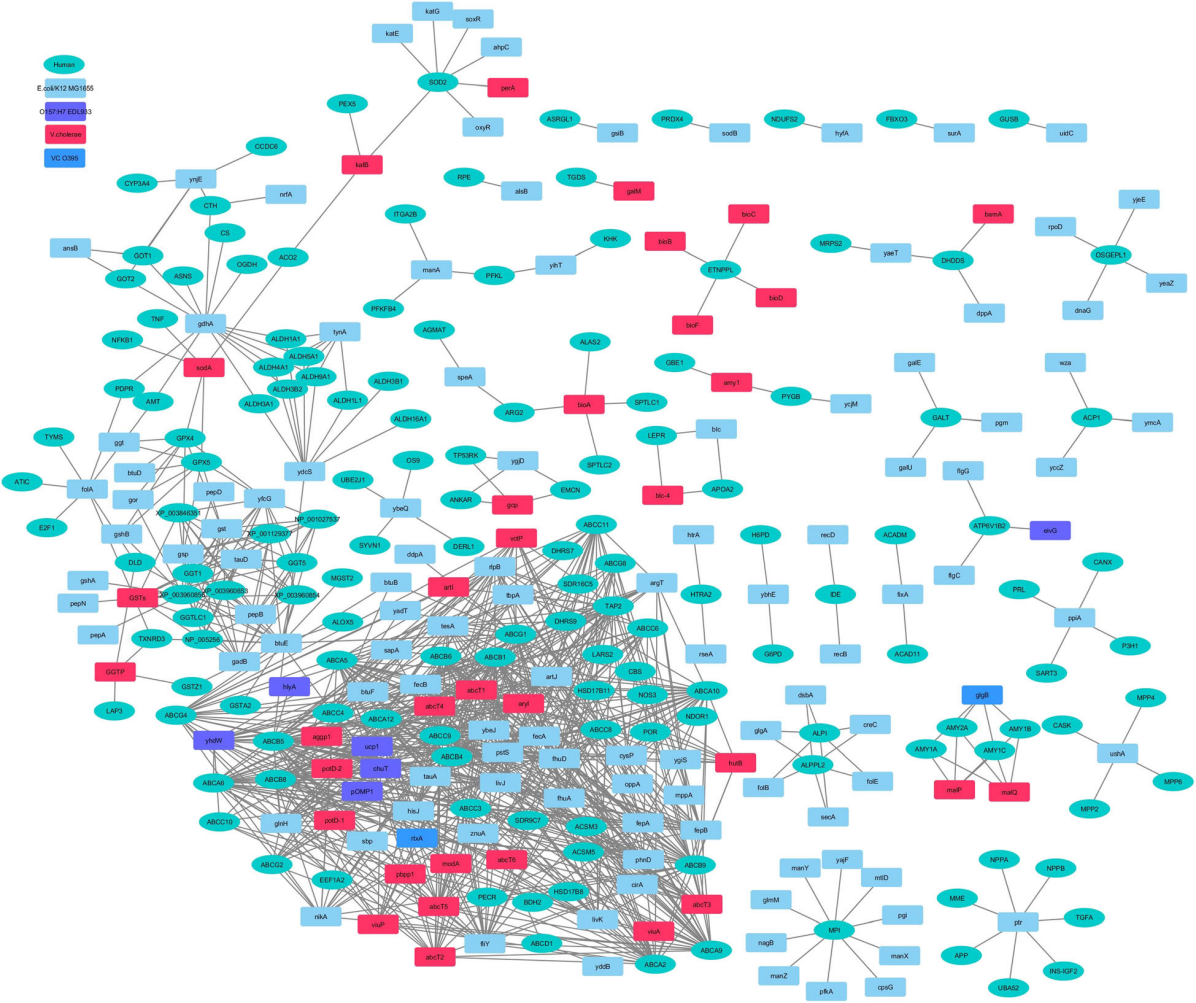
Comparative analysis of the HPI-networks of studied *Escherichia coli* and *Vibrio* strains. Depiction of the differences in the HPI network, along with the involved human (host) and bacterial (pathogen) proteins have been shown. 139 interactions that were common to all the studied stains of *Escherichia coli* and *Vibrio* have not been shown in this figure

The HPI profiles of the studied pathogens consisted of a core set of 122 interactions between the host and pathogen proteins (Fig. 1). Several genera and species-specific interactions were also observed (Figs. 3, 4, 5 and Additional file 3: Figure S1). Some of the observed differences (as compared to *E. coli*) are presented below.

#### Interactions of Shigella (with host) that are unique with respect to interactions of *E. coli* strains (with host)

The HPI profiles pertaining to both *S. dysenteriae* and *S. flexneri* comprised of interactions involving a putative iron transport protein SitA (Fig. 3 and Additional file 3: Figure S2). The putative iron transport gene *SitA* in S. *flexneri* (as well as several commensal enteric bacterial groups) is known to get induced during the intracellular survival stage [39, 40]. Notably, SitA was found to interact with human ATP-dependent mitochondrial porphyrin importer protein (ABCB6), also known for its role in iron metabolic pathways [41]. Given that the in vivo survival of *Shigella* is dependent on acquisition of essential nutrients (such as iron) from the host, the observed interaction between the bacterial putative iron transport protein (SitA) and host ATP-dependent mitochondrial porphyrin importer protein (ABCB6) appears to be important. It may be noted that although SitA was found to be present in the HPI profile of the virulent *Shigella* strains, it is also known to be abundance among non-pathogenic (enteric) bacterial groups [39, 40]. Therefore, this putative iron transport protein probably aids the bacteria in nutrient (iron) acquisition from the host and may not be considered as a ‘pathogenic’ factor [39, 40]. The studied *Shigella* strains were also seen to be involved in 17 additional interactions (Fig. 3 and Additional file 3: Figure S1). Interestingly, while similar interactions were also observed in the pathogenic strain of *E. coli* (EDL933), they were absent in non-pathogenic *E. coli* (K-12 MG1655) (Additional file 3: Figure S2). These 17 HPIs corresponded to the membrane component of amino acid ABC transporter (YhdW). Earlier literatures have indicated at the potential of this membrane component of amino acid ABC transporter to act as a virulent factor. Evidences for the virulence potential of YhdW have previously been shown in different strains of *Shigella* and *E. coli* [5, 42, 43].

#### Salmonella-host interactions that are absent in the *E. coli*-host interactions

The HPI profiles of the studied *Salmonella* strains (Additional file 1) shared as many as 418 HPIs with both pathogenic as well as non-pathogenic *E. coli* strains (Additional file 3: Figure S1). In addition, the three strains of *Salmonella* shared 146 interactions amongst themselves which were absent in the *E. coli* strains (Fig. 4 and Additional file 3: Figure S1). They include interactions pertaining to bacterial proteins like periplasmic murein peptide-binding protein (MppA), para-nitrobenzyl esterase (PnbA), and trifunctional nucleotide phosphoesterase protein (YfkN). Periplasmic murein peptide-binding protein, MppA, which is known to functions as substrate-binding protein, was seen to interact with human proteins involved in translocation of biliary lipids. Pertinently, enteric pathogens including *Salmonella enterica* are known to be resistant to the antibacterial properties of bile and are known to utilize bile salts as nutrients [44]. The para-nitrobenzyl esterase, PnbA in *Salmonella* is known to catalyze the hydrolysis of several beta-lactam antibiotics. It was seen to interact with host carboxylesterases and proteins associated with heparan sulfate proteoglycan (HSPG). Given the multitude of roles played by HSPGs in immunity [45], it is likely that in addition to conferring antibiotic resistance, PnbA could also function in modulating the host immunopathology. Another *Salmonella* protein, namely trifunctional nucleotide phosphoesterase protein YfkN, was found to interact with human proteins associated with nucleotide metabolism and seemed to be involved in the scavenging of nucleotides, particularly under conditions of phosphate shortage [46].

**Fig. 6:**
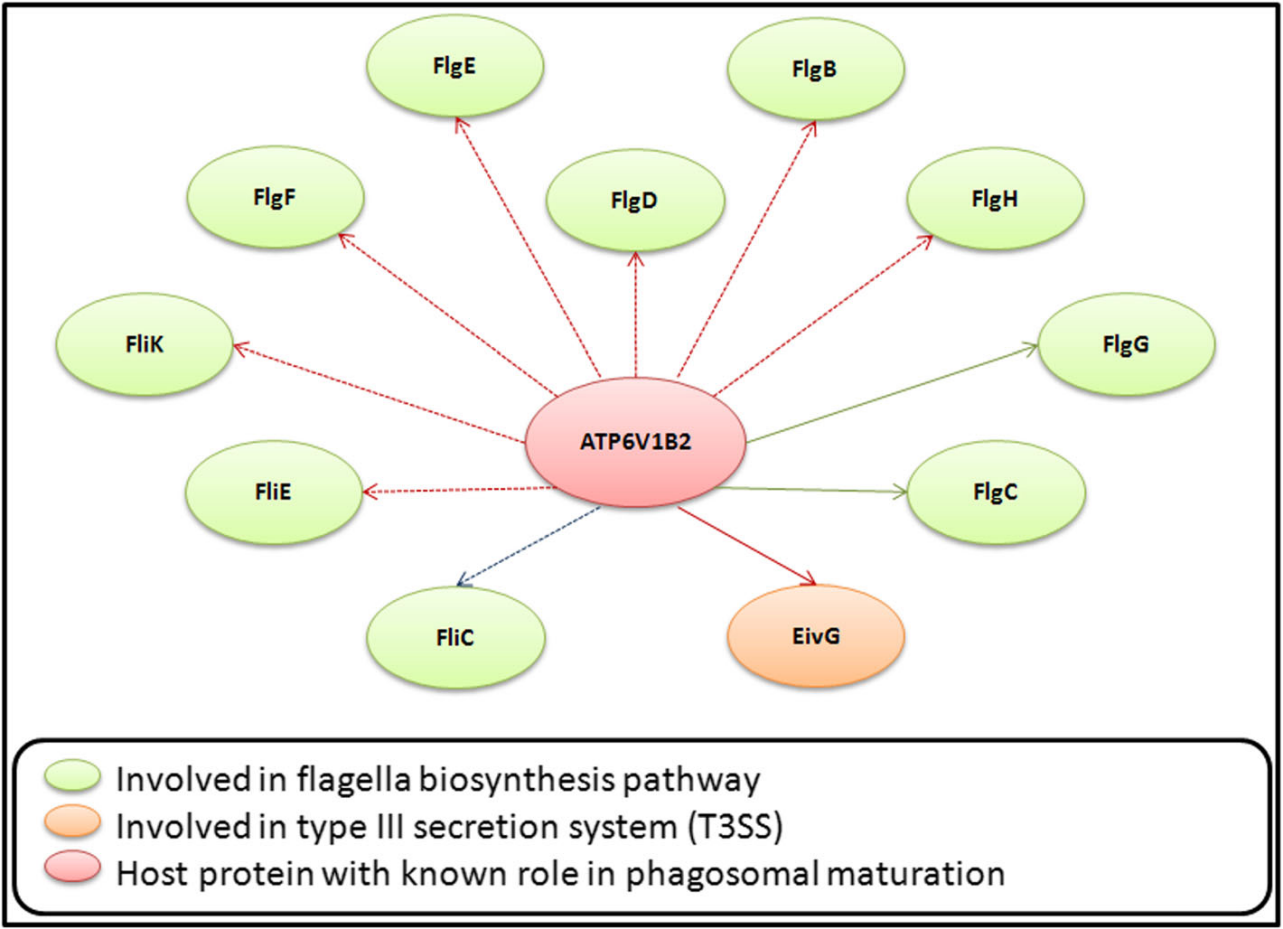
HPIs in *Salmonella* and *E. coli* involving human V-type proton ATPase subunit B (ATP6V1B2). ATP6V1B2 was involved in a total of 11 interactions with the *Salmonella* proteins. 10 of these interactions pertained to the flagella biosynthesis pathways in *Salmonella*. The HPI involving ATP6V1B2 and FliC (blue dashed arrow) was exclusive to *S. enterica* serovar Typhi strains (CT18 and Ty2). The remaining HPI (marked by red solid arrow) which involved EivG/ InvG (type III secretion apparatus protein) was observed in the HPI profile of all the studied *Salmonella* and *E. coli* strains except the non-pathogenic *E. coli* MG1655. Only two of the interactions involving flagella biosynthesis proteins (marked by solid green arrows) were seen in the HPI profiles of the pathogenic *E. coli* strains

Among the human proteins which were involved in this subset of HPIs (with *Salmonella*), V-type proton ATPase subunit B (ATP6V1B2) was found to be most interesting. Our observations indicate an intricate cross-talk between flagella biosynthesis pathway and Type III secretion system (T3SS) in *Salmonella* (Fig. 6). Support for this observation could be obtained from a previous literature which inspected the activation mechanisms of flagella biosynthesis and secretary systems in *Salmonella* [47]*. Salmonella* is known for its ability to arrest phagosomal maturation, thereby re-routing the maturation process towards formation of an invasion vacuole for its survival and replication [48]. Given the role of V-type proton ATPase in phagosomal maturation, it is likely that the re-routing of phagosomal maturation process in *Salmonella* infection is mediated through an ATP6V1B2 dependent process and is likely to include cross-talks between the pathogen’s flagella biosynthesis and T3SS.

#### Host-Vibrio interactions that are absent in Host-*E. coli* interactions

Similar to the HPI profiles in *Shigella* and *Salmonella* strains, the studied strains of *V. cholerae* also exhibited 207 interactions which were not present in the HPI profiles of pathogenic as well as the non-pathogenic *E. coli* strains (Fig. 5 and Additional file 1). The possible biological significance of these interactions is discussed here. *V. cholerae* is known to use multiple strategies to acquire iron for its in vivo survival. This includes utilization of heme from hemoglobin as well as synthesis and transport of vibriobactin [49]. A membrane lipoprotein in *V. cholerae*, namely, ferric vibriobactin-binding protein (ViuP), has previously been shown to function as a transporter for catechol siderophores (like vibriobactin), thereby aiding in iron acquisition [49]. In addition to the human ABC transporters like phosphatidylcholine translocator (ABCB4), ATP-binding cassette sub-family B member 6 (ABCB6), ATP-binding cassette sub-family C member 9 (ABCC9), etc., ViuP was also seen to interact with human antigen peptide transporter 2 (TAP2) (Additional file 3: Figure S3). TAP2, in turn was seen to interact with a host of *Vibrio* proteins, including the ABC transporter ATP-binding proteins (AbcT1, AbcT2, AbcT3, and AbcT4), ferric vibriobactin enterobactin transport system substrate-binding protein (VctP), and periplasmic arginine-binding protein (ArtI) (Additional file 3: Figure S3). Notably, the human antigen peptide transporter 2 (*TAP2*) gene has been previously reported to be up-regulated during bacterial infections [50]. Previously published results coupled with the observations made in this work suggests TAP2 to be an important factor in the *V. cholerae* pathogenic process [49, 50].

#### Host-pathogen interactions that are unique to only *E. coli* strains

Overall, the interaction between proteins from host and those from the studied strains of *Shigella*, *Salmonella* and *Vibrio* were found to contain additional set of interactions which were not observed in the previously reported HPIs involving enteric *E. coli* strains [5]. However, the HPI data corresponding to the former set of organisms also seemed to lack several HPIs which were reported in case of both pathogenic as well as non-pathogenic *E. coli* strains [5]. It was therefore interesting to evaluate whether a subset of these unique interactions is crucial to the *E. coli* infection process. A subset of 18 HPIs was noted to be present only in the HPI profile of *E. coli* O157:H7 EDL933 (Fig. 1). Biological implication of some of these interactions has already been explained in an earlier literature [5]. In particular, HPIs involving bacterial proteins thiosulfate sulfurtransferase (YnjE) and T3SS outer membrane ring protein (EivG) were shown to be critical to the pathogenesis process [5].

### Inter-species variations in interactions of enteric pathogens with host

**Fig. 7:**
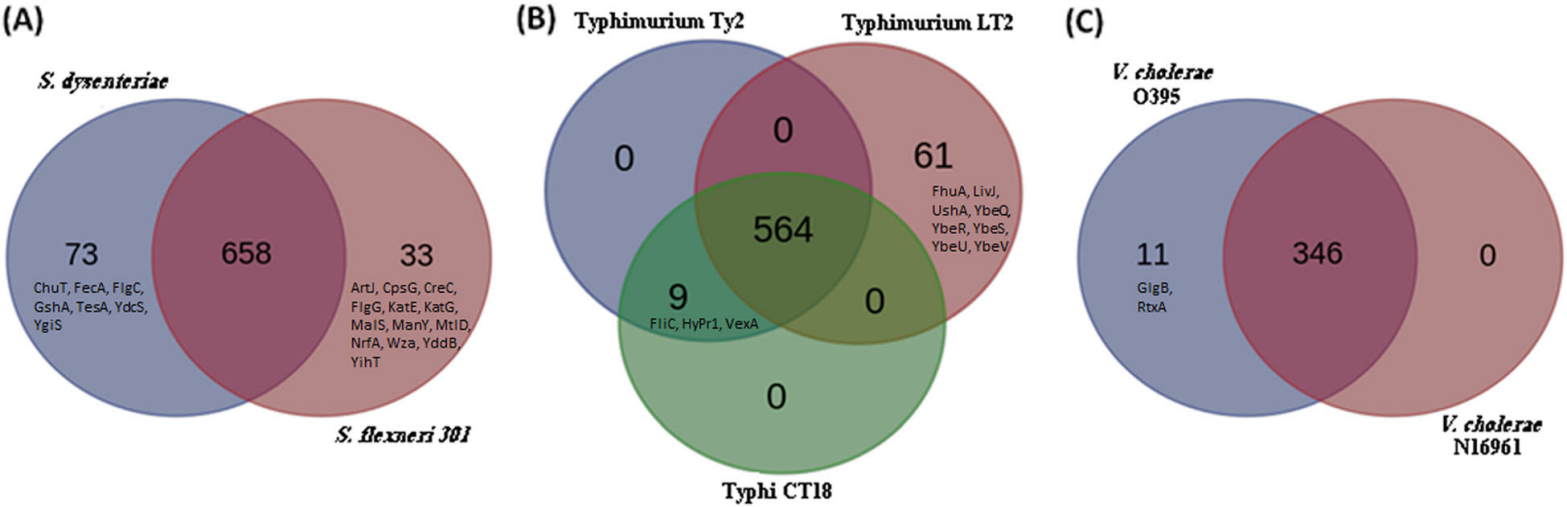
Comparison of intra-species variations in the number of HPIs for the studied pathogens. **a** *Shigella*, **b** *Salmonella*, and **c** *Vibrio*

In addition to genus-specific variations in the HPI profiles of enteric pathogens, several inter-species differences in the HPI profiles were observed (Fig. 7 and Additional file 1). Some of those observed differences are discussed below.

#### Inter-species variations in the HPI profiles of the Shigella strains

While the HPI profiles of both the studied strains of *Shigella* shared majority of the interactions, a few species-specific interactions were also observed (Fig. 7a). A total of 77 HPIs were found to be uniquely present in *S. dysenteriae* when compared with *S. flexneri* 301 (Additional file 1). Among them, HPIs involving acyl-CoA thioesterase I (TesA) were found to be particularly intriguing. Acyl-CoA Thioesterase I was seen to interact with human proteins 7-dehydrocholesterol reductase (DHCR7) and hydroxysteroid 17-Beta Dehydrogenase 11 (HSD17B11) (Additional file 3: Figure S4). While 7-dehydrocholesterol reductase, DHCR7 is an enzyme involved in the conversion of 7-dehydrocholesterol to cholesterol, the hydroxysteroid 17-Beta Dehydrogenase 11, HSD17B11 has been previously reported to be associated with the lipid droplets in the enterocytes [51].

In contrast to the above findings, 33 interactions were seen in the HPI profile of *S. flexneri* 301, which were absent in *S. dysenteriae*. Of them, the most notable were those involving ABC transporter arginine-binding protein 1 (ArtJ). ArtJ in *S. flexneri* 301 was found to be involved in 18 out of the 33 HPIs which were absent in *S. dysenteriae* (Additional file 1). Further, HPIs involving ABC transporter arginine-binding protein 1, ArtJ, were also seen to be present in all the other studied enteric pathogens (except *S. dysenteriae*), suggesting possible role of these interactions in pathogenesis by enteric bacteria. During the infection process, the most probable role ArtJ seems to be binding to arginine and sequestering of arginine from the host [52]. Notably, it was seen to interact with several host ABC transporter proteins. Pertinently, a recent publication which deliberated upon the role of enteric microbes in malnourishment in children indicated possibility of using arginine and glutamine supplements for improving health status [53]. In other words, it was indicated that malnourishment, at least in part, is linked to hijacking of essential nutrients (such as arginine and glutamine) by enteric pathogens, which otherwise feed into key host processes like nucleic acid biosynthesis and cellular replication [53]. The study further stated that malnourishment is also associated with disruption in the host’s ability to transport/ uptake nutrients. The observations made in the present work therefore echoes with reports from earlier literature and provide a possible mechanism through which essential nutrients like arginine are impounded by the enteric pathogens.

#### Inter-species variations in the HPI profiles of the Salmonella strains

Both the serovar Typhi strains (CT18 and Ty2) shared an identical set of proteins which interacted with the host proteins (Fig. 7b). In contrast, the *S. enterica* serovar Typhimurium LT2 demonstrated an additional repertoire of 61 HPIs, which were absent in the serovar Typhi strains. Majority of these interactions in *S. enterica* serovar Typhimurium LT2 were seen to involve one of the three bacterial proteins namely, ferrichrome-iron receptor (FhuA), Leu/Ile/Val-binding protein (LivJ), and UDP-sugar hydrolase (UshA). While FhuA and LivJ are possibly involved in nutrient uptake, UshA has been seen to be beneficial in evading host immune responses [31–33]. Furthermore, the rest of the interactions pertained to a set of poorly characterized bacterial proteins (namely, YbeQ, YbeR, YbeS/ DjlB, YbeU, and YbeV/ DjlC). Of these, YbeS (DjlB) and YbeV (DjlC) have been predicted to be associated to chaperone activity and YbeQ is a Sel1-repeat-containing protein. Notably, the subset of interactions involving YbeQ were also present in three of the five studied *E. coli* strains (EDL933, Sakai, MG1655) as well as the studied *Shigella* strains. It was even more interesting to observe that the aforementioned proteins were encoded in a single gene cassette in *S. enterica* serovar Typhimurium LT2. The only other (studied) organism which encode for the mentioned gene cassette was *E. coli* K-12 MG1655. The above observation when viewed in light of the fact that *S. enteric* serovar Typhimurium strains (which can infect different mammals) are less specific in infecting humans as compared to the serovar Typhi strains [54], it may be assumed that the set of HPIs involving the above mentioned poorly characterized bacterial chaperon and Sel1-repeat-containing proteins play little or no part in infections caused by enteric pathogens to humans. A deeper probe would however be required to establish this hypothesis.

#### Inter-species variations in the HPI profiles of the Vibrio strains

The HPI profiles of *V. cholerae* O395 contained 11 additional interactions as compared to that of *V. cholerae* O1 biovar El Tor N16961 (Fig. 7c). The subset of 11 HPIs involved two bacterial proteins, namely, multifunctional-autoprocessing repeats-in-toxin protein (RtxA), and 1,4-alpha-glucan branching enzyme (GlgB). Given that multifunctional-autoprocessing repeats-in-toxin proteins are known to be present in both the studied strains of *V. cholerae*, the observed differences in HPI profiles were surprising. However, this apparent anomaly could have resulted due to the typical architecture of the RtxA protein. The toxin encoded by different strains of *Vibrio* are known to comprise of conserved and variable domains [55], and the observed variation in the HPI profiles were probably a reflection of the same. With respect to the HPIs involving 1,4-alpha-glucan branching enzyme, GlgB, it may be noted that glycogen is known to play an important role in the survival of *V. cholerae*, especially in nutrient poor aquatic environments [56]. GlgB is known to catalyze the gluconeogenesis process by creating branching of linear glucose chain (through cleavage of 1 → 4 bond and creation of 1 → 6 bond) [57]. It is therefore probable that the observed interactions between the host amylases and the bacterial 1,4-alpha-glucan branching enzymes are a result of the competition for similar substrates between the host and the invading pathogen. Given the fact that El Tor strains of *V. cholerae* (like N16961) are known to be more virulent than the classic strains (like O395) [58], the above observations may seem to be counter-intuitive. However, it may be noted that classical biotypes of *V. cholerae* have been observed to attain a viable but non-culturable (VBNC) state on co-culturing with El Tor biotypes [59]. It is therefore likely that in addition to the cross-talks with the host, closely related biotypes of *V. cholerae* may also interact among themselves. The outcome of the *V. cholerae* infection process is therefore not only dependent on its interaction with the host but is also influenced by the cross-talks among the infecting biotypes/ strains.

## Discussion

Microbes which grow in similar ecological niche environments (e.g. inside human gut) are often seen to share a lot of common features that are essential to adapt to that environment [60]. Results presented through the in silico findings of this work also indicate a common repertoire of protein-protein interactions which were seen to be omnipresent in the host-pathogen interaction profiles of the studied pathogens belonging to genus *Escherichia*, *Shigella*, *Salmonella* and *Vibrio*. This subset of HPIs comprised of 122 interactions involving 122 host and 17 bacterial proteins. These 17 bacterial proteins may be considered as ‘core factors’ probably responsible for the bacteria to infect the host. However, these 122 interactions were also seen to be present in the HPI profile of the studied non-pathogenic *E. coli* strain. Hence, these set of HPIs should probably be regarded as ‘niche’ factors (rather than terming them as virulence factors), which provide an adaptive advantage to the inhabiting organisms inside the host. For example, ABC transporter periplasmic-binding protein, SapA, has previously been shown to be involved in the neutralization of antimicrobial peptides (AMPs), thereby aiding opportunistic pathogens like *Haemophilus influenzae* to infect the host [34]. Although previous literatures have linked the activity of SapA to pathogenesis, the current analysis indicates its participations even in the PPI networks involving non-pathogenic bacteria (*E. coli* MG1655). SapA therefore appears to be a more generic niche factor which is essential for the survival of most (enteric) bacterial species inside its host.

**Fig. 8:**
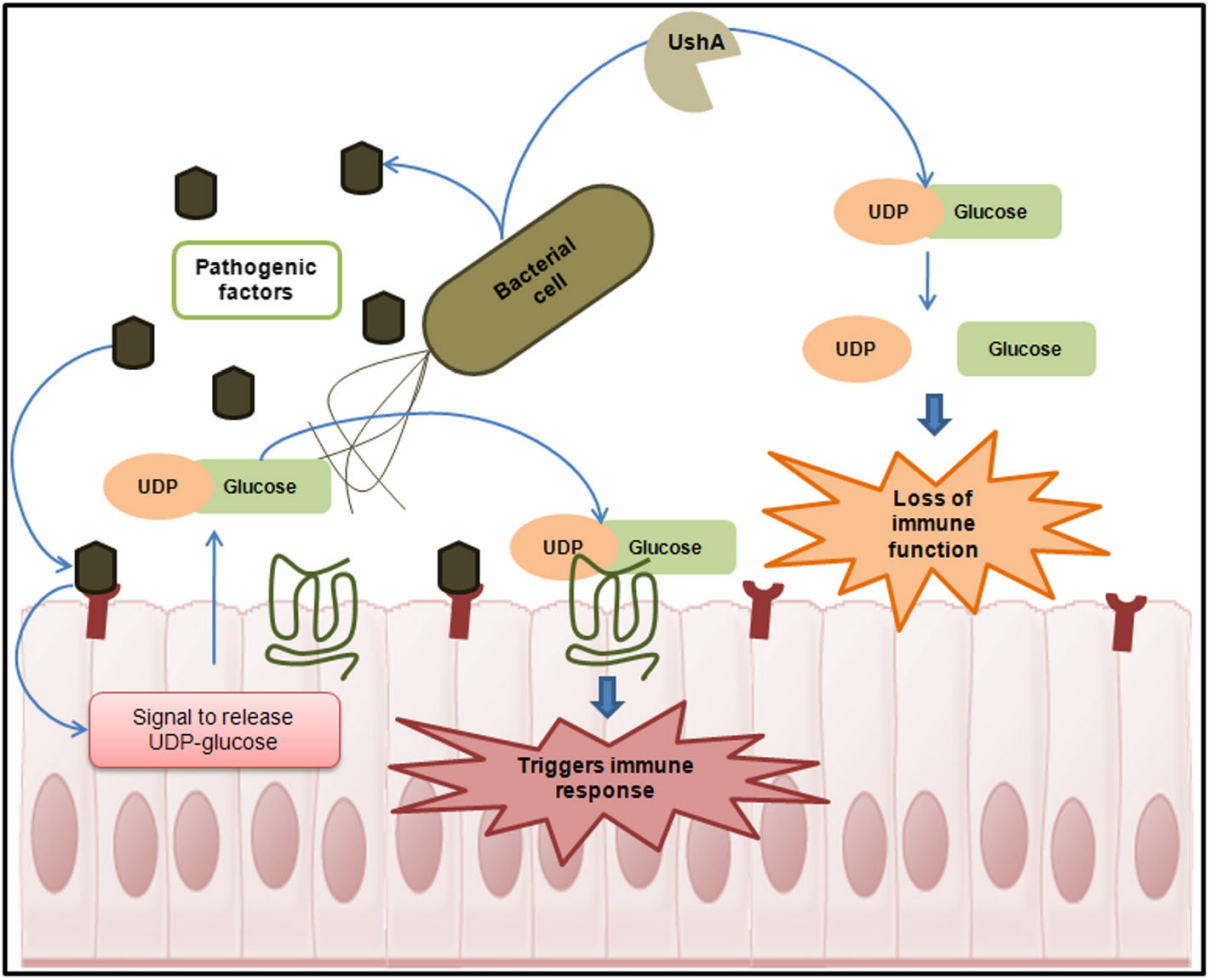
Role of UDP-glucose in innate immune response and the probable UshA mediated mechanism of evading host defense by invading enteric pathogens. Human epithelial cells, in response to bacterial infection, release UDP-glucose. The released UDP-glucose acts as a signal to trigger innate immune responses. UshA, a UDP-glucose hydrolase can degrade the signaling molecule (UDP-glucose), and possibly acts as a virulence factor by abrogating the host defenses

The HPI networks of the studied pathogens were seen to comprise of a few high degree nodes. Such nodes are expected to be involved in crucial biological functions. In other words, presence/ absence of such high degree nodes in the HPI network might translate to important variations in the infection processes. UDP-sugar hydrolase (UshA) and acyl-CoA thioesterase I (TesA) were identified as two such bacterial proteins which had high degree of interactions in the HPI network but was selectively absent in one or a few studied strains. UshA was found to be consistently present in the HPI profiles of studied bacterial strains, except in the two strains of *S. enterica* serovar Typhi. Based on previously published literature [31–33], we hypothesize that UshA may be involved in the hydrolysis of UDP-glucose. UDP-glucose and its receptor P2RY14 have previously been shown to be key players in triggering innate mucosal immune responses [61]. UshA may thus be associated with the capability to inhibit the innate immune system, thereby facilitating the bacterial infection process (Fig. 8). The absence of UshA in HPI-network (of *S. enterica* serovar Typhi strains) is indicative of possibly a different pathogenic mechanism in *S. enterica* serovar Typhi strains. It is probable that like in a few other gram negative intracellular pathogens [62], *S. enterica* serovar Typhi strains may be utilizing UDP-glucose to sequester energy in the nutrient limited environment within the vacuole.

Similarly, while acyl-CoA thioesterase I (TesA) was found to be absent in the HPI profile of *Shigella flexneri* 301, it was present in most other bacteria (including *Shigella dysenteriae*). TesA is a multifunctional enzyme that has thioesterase, lysophospholipase and protease activities [63–65] and inactivation/ absence of TesA has previously been reported to be associated with increased drug susceptibility and lipid metabolism [66]. TesA was seen to interact with human DHCR7 (7-dehydrocholesterol reductase), an enzyme involved in the conversion of 7-dehydrocholesterol to cholesterol (Additional file 3: Figure S4). Further, TesA was also seen to interact with human hydroxysteroid 17-Beta Dehydrogenase 11 (HSD17B11), which has been previously reported to be associated with the lipid droplets in the enterocytes [51]. While, the *tesA* gene in *S. flexneri* is known to be non-functional [67], it is pertinent to note that the initial stages of infection caused by most enteric pathogens (including *Shigella*) are often dependent on cholesterol and sphingolipids which are possibly acquired from the host enterocytes [5, 68]. The above findings are possibly suggestive of the presence of an alternate functional machinery in *S. flexneri* that enables the pathogen to form lipid rafts to escape host defenses as well as attain resistance to antibacterial agents. However, since this is an in silico finding, additional experimental data would be required to validate this hypothesis.

In addition to the core set of HPIs, different groups of bacteria were seen to diverse strategies to best exploit the resources within the host micro environment. Interactions pertaining to a putative iron transport protein (SitA) in *Shigella*, a nucleotide phosphoesterase (YfkN), a para-nitrobenzyl esterase (PnbA) and a periplasmic peptide-binding protein (MppA) in *Salmonella* and ABC transporter ATP-binding proteins (AbcT1, AbcT2, AbcT3) in *Vibrio* are a few worth mentioning in this regard. While SitA in *Shigella*, MppA and YfkA in *Salmonella* and AbcTx in *Vibrio* were seen to be involved in acquisition of nutrients (like iron and phosphate) from the host, PnbA was seen to play roles in antibiotic resistance and immune modulation in *Salmonella* infection.

Given that these proteins do not have any homologues in humans, these bacterial proteins assume importance from the perspective of development of directed therapeutic strategies. Bacteria are more prone to developing (or acquiring) resistance to broad spectrum antibiotics, as compared to narrow spectrum antibiotics. In part, this could be attributed to horizontal transfer of genes within bacterial groups residing in a close association. Consequently, the strategy to target a set of proteins which are unique to small groups of bacteria and/ or are involved in interaction with the host in specific groups of bacteria is expected to reap benefits in the long run. The bacterial protein membrane component of amino acid ABC transporter (YhdW), which has previously been implicated in the pathogenicity of *Shigella* and *E. coli* [5, 42, 43], and did not have any homologues in humans seemed to be a prospective therapeutic candidate against drug-resistant strains of *Shigella* and *E. coli*. Given that most of these proteins (which are involved in interaction with the host) are associated with the bacterial cell surface, the activity of these proteins can be restricted/ mitigated with relative ease using one or a combination of drug molecules.

It was also interesting to observe differences between the HPI profiles of (a) enteric pathogens as compared to a lung pathogen, and (b) gram positive and gram negative bacteria (see Additional file 3: Results section). In general, a higher number of proteins in gram negative bacteria were seen to be involved in interacting with the host proteins as compared to those in gram positive bacteria. For example, only 30 *Mycobacterium tuberculosis* H37Rv (*Mtb*) proteins were seen to interact with the host as compared to over a 100 (between 102 and 122) in most of the studied gram negative strains. Further, there was no significant overlap in set of bacterial proteins involved in HPI among the lung and the enteric pathogens. Only four bacterial proteins namely, gamma-glutamyltransferase (Ggt), vitamin B12 import ATP-binding protein (BtuD), hydrogenase-4 component A (HyfA) and glucose-6-phosphate isomerase (Pgi) were found to occur in both the networks. When compared to the gram negative bacteria, the gram positive bacteria residing in the human gut were seen to share fewer interactions with the host. The above observations may partially be attributed to the higher number of ATP-binding cassette transporters (ABC transporters) that are involved in interaction with the host in gram negative bacteria. The ABC transporters are involved in the active (energy driven) movement of molecules across the inner and outer membranes of the cell. Our observations concord with previous reports which suggested the occurrence of a higher number of surface proteins involved in macromolecule transport in gram negative bacteria as compared to their gram positive counterparts [69, 70]. The outer membrane of gram negative bacteria which harbors several of the above mentioned proteins is also absent in gram positive bacteria [69, 70], thus explaining our observation of a higher number of HPI associated proteins in gram negative bacteria as compared to gram positive ones.

The HPIs reported in this work and the hypothesis derived from them were inferred on the basis of an in silico approach. Results obtained from such an in silico approach may contain a few false positive outcomes. Therefore, the confidence of the predicted HPIs (and their proposed mechanisms of actions) could have been improved if the method of predicting the HPIs was augmented with experimental evidences (gene/ protein/ metabolite expression data). The procedure had been adopted in one of the earlier works pertaining to the study of the survival of *Mtb* inside the human body [6]. However, the lack of suitable host and bacterial gene/ protein/ metabolite expression data prevented us from adopting the strategy in this case.

Further, the study identifies a few key bacterial proteins which may act as prospective therapeutic targets. While we have ascertained that these proteins do not share any homology with human proteins (data not shown), we could not test the essentiality of these bacterial gene products during in vivo survival, due to lack of adequate data. While popular knowledgebases like Database of Essential Genes (DEG) provide some information regarding gene essentiality, most of the data pertains to growth under rich nutrient medium which do not mimic the in vivo growth conditions prevalent inside a host.

In addition to HPIs, other virulent factors such as the bacteriotoxins may contribute to the pathogenesis of a disease-causing bacteria. Examples of such molecules include shiga toxins in *E. coli* and *Shigella strains*, choleragens in *V. cholerae* which can impact the degree of pathogenicity caused by the microbe. Cross-talks between the host and the pathogen involving such virulent factors were beyond the scope of this manuscript.

## Conclusion

In spite of certain limitations, the current in silico study, possibly for the first time highlight a comparative analysis of HPI among different gut associated bacterial groups. Results presented herein provide insights into the bacterial processes that are possibly involved in the survival/ adaptation of various enteric pathogens inside the host body. The bacterial proteins which have been identified to be involved in interaction with host proteins (especially those which demonstrated high centrality measures in the HPI networks) could serve as attractive candidates for rational drug designing, thereby helping to tackle the menace of antibiotic resistance among bacterial pathogens.

## Methods

**Fig. 9:**
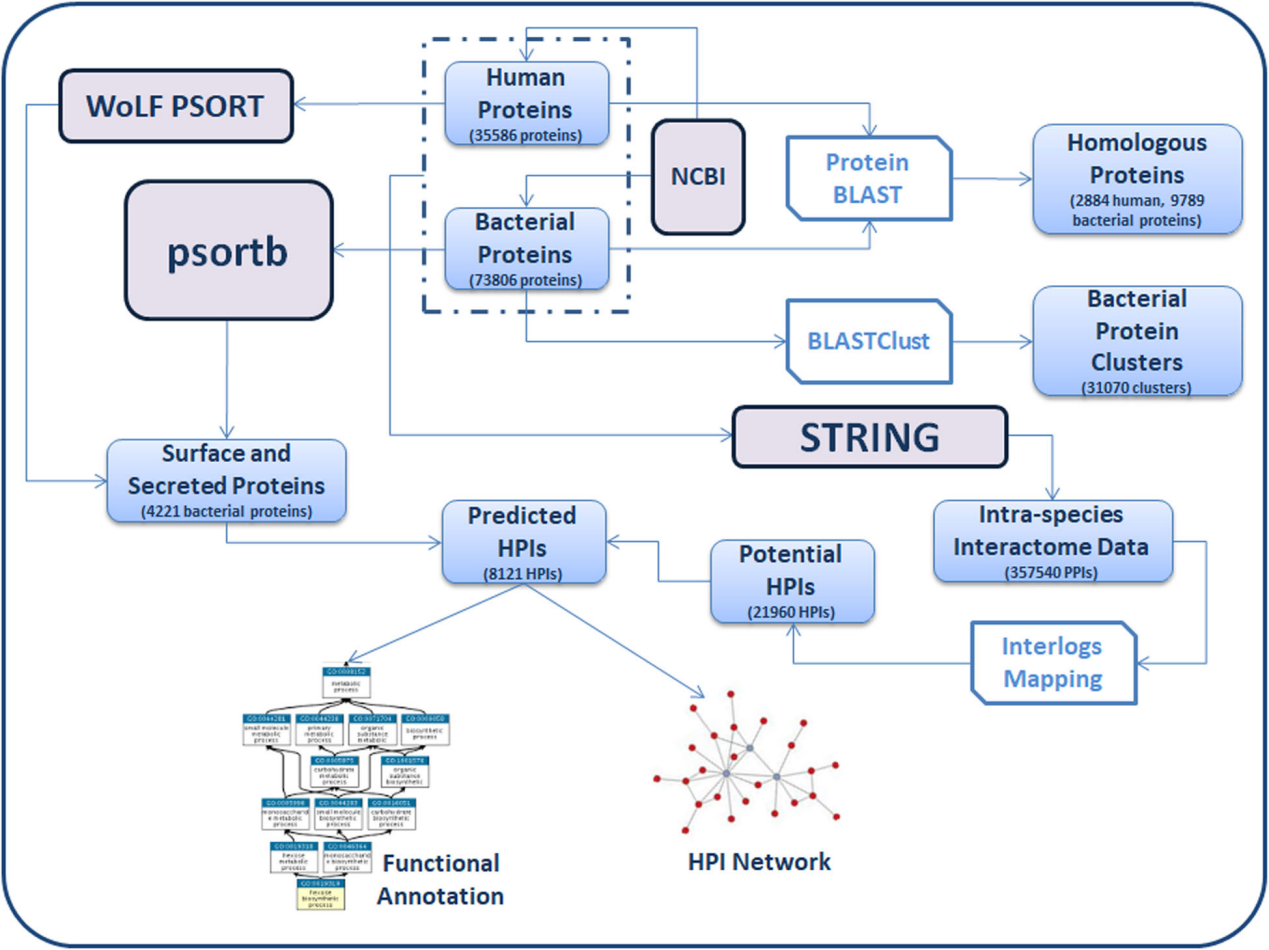
Schematic representation of the methodologies adopted for the study

The methodology adopted for identifying interacting host-pathogen protein pairs is similar to that used in our earlier published study [5]. The method is schematically depicted in Fig. 9 and details of the method have been provided in the Additional file 3: Materials and Methods section. In brief, human and bacterial protein sequences were first obtained from NCBI database (Additional file 3: Appendix 2). Using the BLASTClust program, a total of 16,599 unique clusters were identified among the bacterial protein sequences. Homologies between human and bacterial proteins were determined using BLASTp analysis. The most probable sub-cellular localization for the host and bacterial protein sequences were inferred using WoLF PSORT [71] and PSORTb version 3.0.2 [72] respectively. The HPIs were derived from information pertaining to (a) the template intra-species interaction data available from STRING database, version 9.1 [73] (http://string-db.org/) and (b) identified homology among human and bacterial proteins, (c) clustering of bacterial proteins, and (d) inferred sub-cellular localization of host and bacterial proteins. Subsequently, the host–pathogen interaction protein pairs were collated together to form HPI networks for each of the studied organisms and were analyzed for network properties using Cytoscape (version 2.8) [74] and CompNet [75]. The functional analysis of the proteins involved in HPIs was performed in terms of (i) Gene Ontology (GO) enrichment analysis, and (ii) KEGG functional pathway analysis. The GO enrichment analysis was performed using the data analysis module of STING web resource [76] (http://string-db.org/).

## Supplementary information

**Additional file 1.** List of predicted Host Pathogen Interactions (HPIs) for the studied host-pathogen models.

**Additional file 2.** Network statistics for each of the human and bacterial proteins which were associated to the host-pathogen interaction (HPI) network.

**Additional file 3.** Supplementary Methods, Results and Images.

**Additional file 4.** Gene Ontology analysis of the human and bacterial proteins associated to the host-pathogen interaction (HPI) network.

## Data Availability

The data sets generated and/or analysed in this study can be found in The National Center for Biotechnology Information database using the accession numbers listed in Additional file 3: Appendix 2.

## Abbreviations

ABCB4: ABC transporters like phosphatidylcholine translocator ABCB4
ABCB6: ATP-binding cassette sub-family B member 6
ABCC9: ATP-binding cassette sub-family C member 9
ACP1: Acid phosphatase 1
AMPs: Antimicrobial peptides
ArtI: Arginine-binding periplasmic protein 1
ATP6V1B2: V-type proton ATPase subunit B
BtuD: Vitamin B12 import ATP-binding protein
DEG: Database of Essential Genes
DHCR7: 7-dehydrocholesterol reductase
EivG: Type III secretion system outer membrane ring protein
FhuA: Ferrichrome-iron receptor
Ggt: Gamma-glutamyltransferase
GlgB: 1,4-alpha-glucan branching enzyme
Gor: Glutathione reductase
GshB: Glutathione synthetase
HPI: Host-Pathogen Protein-Protein Interaction
HSD17B11: Hydroxysteroid 17-Beta Dehydrogenase 11
HSPG: Heparan sulfate proteoglycan
HyfA: Hydrogenase-4 component A
LivJ: Leu/Ile/Val-binding protein
MppA: Periplasmic murein peptide-binding protein
MTB: *Mycobacterium tuberculosis* H37Rv
NLR: NOD-like receptor
OSGEPL1: O-sialoglycoprotein endopeptidase like 1
PAMPs: Pathogen-associated molecular patterns
PepA: Cytosol aminopeptidase
PepD: Cytosol non-specific dipeptidase
PepN: Aminopeptidase N
Pgi: Glucose-6-phosphate isomerase
PnbA: Para-nitrobenzyl esterase
PPI: Protein-Protein Interaction
ROS: Reactive oxygen species
RtxA: Multifunctional-autoprocessing repeats-in-toxin protein
SapA: ABC transporter periplasmic-binding protein
SitA: Putative iron transport protein SitA
SodB: Superoxide dismutase
T3SS: Type III secretion system
TAP2: Antigen peptide transporter 2
TbpA: Transferrin binding protein A
TesA: Acyl-CoA thioesterase I
UshA: UDP-sugar hydrolase
VBNC: Viable but non-culturable
ViuP: Ferric vibriobactin-binding protein
WHO: World Health Organization
YfkN: Trifunctional nucleotide phosphoesterase protein
YhdW: Amino acid ABC transporter
YnjE: Thiosulfate sulfurtransferase
