# Supplementary material for: Investigating host-bacterial interactions among enteric pathogens
Source: BMC Genomics. 2019 Dec 27;20:1022. doi: 10.1186/s12864-019-6398-2 (PMC6935094; doi:10.1186/s12864-019-6398-2)
Supplement: Supplementary file 3 — Additional file 3. Supplementary Methods, Results and Images. [file 12864_2019_6398_MOESM3_ESM.docx]

**Investigating host-bacterial interactions among enteric pathogens**

**Tungadri Bose,^1,2#^ K. V. Venkatesh,^2^ and Sharmila S. Mande^1,*^**

^1^Bio-Sciences R&D Division, TCS Innovation Labs, Tata Consultancy Services Limited, Pune, India

^2^Department of Chemical Engineering, Indian Institute of Technology Bombay, Mumbai, India

*Corresponding Author

^#^Registered for PhD in Department of Chemical Engineering, Indian Institute of Technology Bombay, Mumbai, India

**Materials and Methods**

**Data Acquisition and Preprocessing**

First, the protein sequences for human and the studied bacterial strains were downloaded from the NCBI database (<ftp://ftp.ncbi.nlm.nih.gov/genomes/>). Accession details of the genomes analyzed in this study are provided in Appendix 2. Orthologous groups of proteins were identified among the studied bacterial strains using BLASTClust program from the standalone BLAST package. A threshold criteria of 95% identity and 80% overlap was applied to identify 16599 unique clusters of orthologous bacterial proteins. Further, homology between human and bacterial proteins was also ascertained using BLASTp analysis. BLASTp results having e-value lower than 1 × e^−10^ were further filtered using a criteria of 30% sequence identity and 80% sequence overlap. The template interactions library was built using the intra-species interactome data of human and the studied bacterial strains. The intra-species interactome data was obtained from the STRING database, version 9.1 (Franceschini et al., 2013) (<http://string-db.org/>). The intra-species interactions in the template library were processed in accordance to the method described in our earlier work (Bose et al., 2017) to obtain a library of high confidence intra-species PPIs.

**Identification of Sub-cellular Localization of Proteins**

It was assumed that the host-pathogen interactions would be manifested by the host and bacterial proteins which were localized either on the cell surface or is secreted outside the cell. Consequently, the host and bacterial protein sequences were screened for their sub-cellular localization using established bioinformatic approaches. While PSORTb version 3.0.2 (Gardy et al., 2005) was employed for the analysis of bacterial proteins, WoLF PSORT (Horton et al., 2007) was used for predicting the sub-cellular localization of the human proteins.

**Prediction of Protein–Protein Interactions between host and bacteria**

The methodology for predicting PPIs between the host (human) and bacterial proteins was borrowed from our earlier work concerning the study of host-*E. coli* PPIs (Bose et al., 2017). Briefly, for any given intra-species template PPI (either bacterial or human), a host-bacterial PPI was assumed to occur if a homologue of at-least one of the interacting proteins existed in the other organism (human or bacteria). The potential host-bacterial PPIs were further screened for cellular co-localization of the participating proteins. Subset of identified interactions wherein the bacterial protein was either secreted or localized on the cell membrane or cell wall and the host protein was present on the cell surface (or exterior to it) were considered as the final host-bacterial interacting pairs.

**Host–Bacteria Interaction Network Analysis**

The host–pathogen interaction protein pairs as obtained from the earlier analysis were collated together to form HPI networks for each of the studied organisms. Subsequently, each of the interaction networks was analyzed for network properties like degree and betweenness centralities of each node (constituent proteins in the network). For the purpose, the host–pathogen interactome data for each of the studied organism were analyzed using Cytoscape (version 2.8) (Shannon et al., 2003), a widely used platform for network analysis and visualization. In addition, CompNet (Kuntal et al., 2016), a tool for comparative analysis of multiple biological networks, was also used for a comparative analysis of the HPI networks.

**Functional Analysis**

The functional analysis performed in this study comprised of (i) Gene Ontology (GO) enrichment analysis, and (ii) KEGG functional pathway analysis. The GO enrichment analysis was performed using the data analysis module of STING web resource (Szklarczyk et al., 2015) (<http://string-db.org/>). For the purpose, the minimum required interaction score was considered to be 0.9. Further the maximum number of interactors to show at 1^st^ shell was set to 5 for bacteria proteins and 0 for human proteins. The maximum number of interactors to show at 2^st^ shell was always set to 0. GO terms qualifying a minimum gene count of two with a false discovery rate (FDR) <0.001 were considered. It may be noted that DAVID (Huang et al., 2009), the most popular tool for GO enrichment analysis was not used in the current study. The choice of STRING over DAVID was governed by the fact that unlike STRING, all the studied bacterial organisms were not listed in the back-end database of DAVID, thereby making the adoption of the tool infeasible for the current study.

The KEGG pathway analysis was performed to analyze the relevance of the predicted human interactors in the bacterial infection processes. In particular, the human infection pathways pertaining to those caused by *E. coli*, *Shigella* and *Salmonella* were checked. The set of human proteins listed in each of these pathways were probed for association with a set (or a sub-set) of proteins involved in HPIs. Precisely, the connectivity between the human proteins involved in HPIs (along with their 1^st^ degree neighbors) and the human proteins reported in the KEGG infection pathways were studied. The shortest paths were ascertained on the basis of the human interactome data (minimum confidence score of 0.9), as obtained from the STRING database. The sub-set of human proteins from the KEGG infection pathways were considered to be associated to the HPI network if they were connected to the human HPI components by a maximum path-length of two.

**Results**

**Comparison of Host-Pathogen interactions involving gram positive and gram negative bacteria**

It may be noted that all the enteric pathogens whose HPI profiles were analyzed in this work were gram negative in nature. The choice of the organisms was in part governed by the fact that most enteric pathogens are gram negative in nature (WHO | WHO publishes list of bacteria for which new antibiotics are urgently needed, 2018). Further, the rapid emergence of antibiotic traits among the gram negative inhabitants of the gut also led us to the choice of the study organisms. However, it would be interesting to understand how the set of HPIs pertaining to gram negative bacteria compares to those of gram positive organisms. A brief overview in this regard is discussed below.

The comparison between the HPI profiles of gram positive and gram negative pathogens was performed using data from one of our earlier publications, wherein HPIs between human and *Mycobacterium tuberculosis* (*Mtb*) was analyzed (Refer to MTB HPI paper). The *Mtb* HPI network comprised of 385 interactions involving 317 host and 34 bacterial proteins (Bose et al., 2018). The number of observed interactions in *Mtb* was seen to be considerably lesser than most of the studied gram negative pathogens, except for the studied strains of *Vibrio*, where the number of HPI were comparable. Notably, the number of *Mtb* proteins involved in interactions with the host was found to be significantly less than any of the gram negative pathogens. More importantly, there was an overlap of only four proteins between the proteomes of *Mtb* and the studied gram negative pathogens that were involved in interaction with the host. These proteins included bacterial gamma-glutamyltransferase (Ggt), vitamin B12 import ATP-binding protein (BtuD), hydrogenase-4 component A (HyfA) and glucose-6-phosphate isomerase (Pgi). Therefore, the mechanism of virulence between gram positive and gram negative bacteria seemed to be significantly different. However, the observed difference in the HPI profiles could also be due to other confounding factors, such as the site of infection/ localization of the pathogen. Although *Mtb* is known to inhabit host's enteric channel as well as in bone marrow, the pathogen is primarily known to infect the lung. To ascertain whether the HPI profiles between gram positive and gram negative bacteria were indeed different or the observed variations were due to the differences in the site of inhabitation of the bacteria, the HPI profiles for three gram positive enteric bacteria were also studied.

Of them, the first was *Bacillus cereus* ATCC 14579, an opportunistic gram positive enteric bacterium, which is known to mainly associated with food poisoning. Its HPI profile constituted of 198 interactions involving 105 human and 30 bacterial proteins. The second, *Bifidobacterium adolescentis* ATCC 15703 is a commensal inhabitant of the healthy human intestinal tract. It participated in 107 interactions with 34 host proteins through 22 proteins encoded in its genome. The third organism, *Lactobacillus acidophilus* 30SC usually resides in the human gastrointestinal tract where it ferments glucose into lactic acid and are known for its probiotic properties. It interacted with the host through 15 interactions involving only nine host and an equivalent number of bacterial proteins. These observations indicated that the gram positive bacteria (irrespective of their pathogenic nature/ severity) indeed participated in fewer interactions with the host as compared to gram negative bacteria. It further hinted at the possibility that bacteria residing in the anterior part of the alimentary canal to be involved in a higher number of interactions with the host as compared to those residing at the posterior end of the alimentary tract, although deeper inspection would be required to establish the fact. Further, in concordance with our earlier observations involving the *Mtb* HPI profile, the set of bacterial proteins interacting with the host in gram positive enteric bacteria had no resemblance with the corresponding set from gram negative enteric bacteria. More importantly, there was no overlap between the bacterial proteomes (involved in interaction with the host) for the three studied species of gram positive enteric bacteria as well as *Mtb*.

Overall, the observations seemed to indicate that gram positive and gram negative groups of bacteria employ distinctly different strategies to interact with the human host. Further, the mode of interaction also seemed to be dependent on the site of inhabitation/ infection of the bacterial species.

**References**

Bose, T., Das, C., Dutta, A., Mahamkali, V., Sadhu, S., and Mande, S. S. (2018). Understanding the role of interactions between host and Mycobacterium tuberculosis under hypoxic condition: an in silico approach. *BMC Genomics* 19, 555. doi:10.1186/s12864-018-4947-8.

Bose, T., Venkatesh, K. V., and Mande, S. S. (2017). Computational Analysis of Host-Pathogen Protein Interactions between Humans and Different Strains of Enterohemorrhagic Escherichia coli. *Front. Cell. Infect. Microbiol.* 7, 128. doi:10.3389/fcimb.2017.00128.

Franceschini, A., Szklarczyk, D., Frankild, S., Kuhn, M., Simonovic, M., Roth, A., et al. (2013). STRING v9.1: protein-protein interaction networks, with increased coverage and integration. *Nucleic Acids Res.* 41, D808–D815. doi:10.1093/nar/gks1094.

Gardy, J. L., Laird, M. R., Chen, F., Rey, S., Walsh, C. J., Ester, M., et al. (2005). PSORTb v.2.0: expanded prediction of bacterial protein subcellular localization and insights gained from comparative proteome analysis. *Bioinforma. Oxf. Engl.* 21, 617–623. doi:10.1093/bioinformatics/bti057.

Horton, P., Park, K.-J., Obayashi, T., Fujita, N., Harada, H., Adams-Collier, C. J., et al. (2007). WoLF PSORT: protein localization predictor. *Nucleic Acids Res.* 35, W585-587. doi:10.1093/nar/gkm259.

Huang, D. W., Sherman, B. T., and Lempicki, R. A. (2009). Bioinformatics enrichment tools: paths toward the comprehensive functional analysis of large gene lists. *Nucleic Acids Res.* 37, 1–13. doi:10.1093/nar/gkn923.

Kuntal, B. K., Dutta, A., and Mande, S. S. (2016). CompNet: a GUI based tool for comparison of multiple biological interaction networks. *BMC Bioinformatics* 17, 185. doi:10.1186/s12859-016-1013-x.

Shannon, P., Markiel, A., Ozier, O., Baliga, N. S., Wang, J. T., Ramage, D., et al. (2003). Cytoscape: a software environment for integrated models of biomolecular interaction networks. *Genome Res.* 13, 2498–2504. doi:10.1101/gr.1239303.

Szklarczyk, D., Franceschini, A., Wyder, S., Forslund, K., Heller, D., Huerta-Cepas, J., et al. (2015). STRING v10: protein-protein interaction networks, integrated over the tree of life. *Nucleic Acids Res.* 43, D447-452. doi:10.1093/nar/gku1003.

WHO | WHO publishes list of bacteria for which new antibiotics are urgently needed (2018). *WHO*. Available at: http://www.who.int/mediacentre/news/releases/2017/bacteria-antibiotics-needed/en/ [Accessed April 16, 2018].

**Additional Figures**

**Figure S1:** Euler diagram representing the commonalities and differences between number of HPIs involving human proteins and the studied *Escherichia coli* strains with respect to the studied (A) *Shigella*, (B) *Salmonella*, and (C) *Vibrio* strains.

**
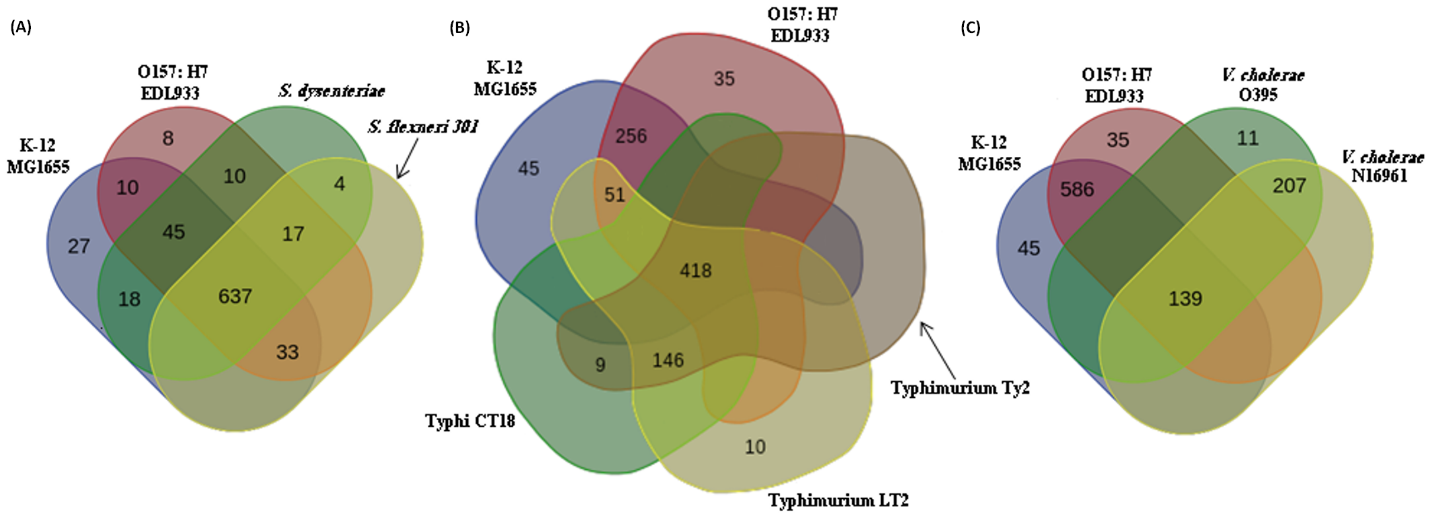
**

**Figure S2:** Visualization of the HPIs in the studied *Shigella* strains that were absent in non-pathogenic *E. coli* strains. Interactions involving SitA (red edges) are also absent in pathogenic strains of *E. coli*.


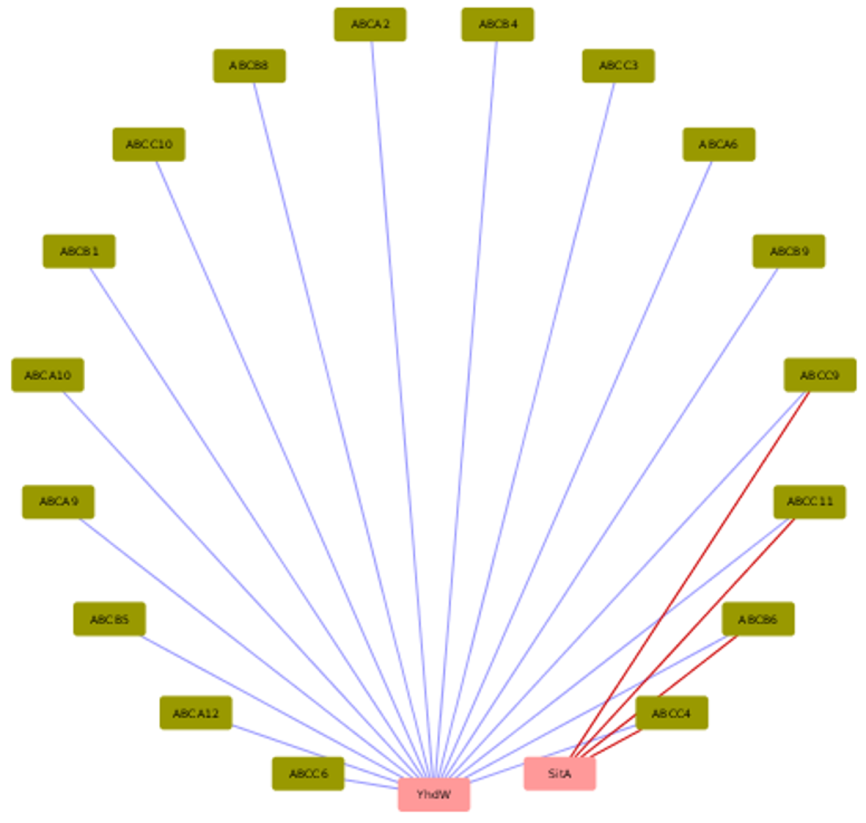


**Figure S3:** Visualization of the *V. cholerae* associated HPIs that were seen to be absent in the HPI profile of *E. coli* strains.


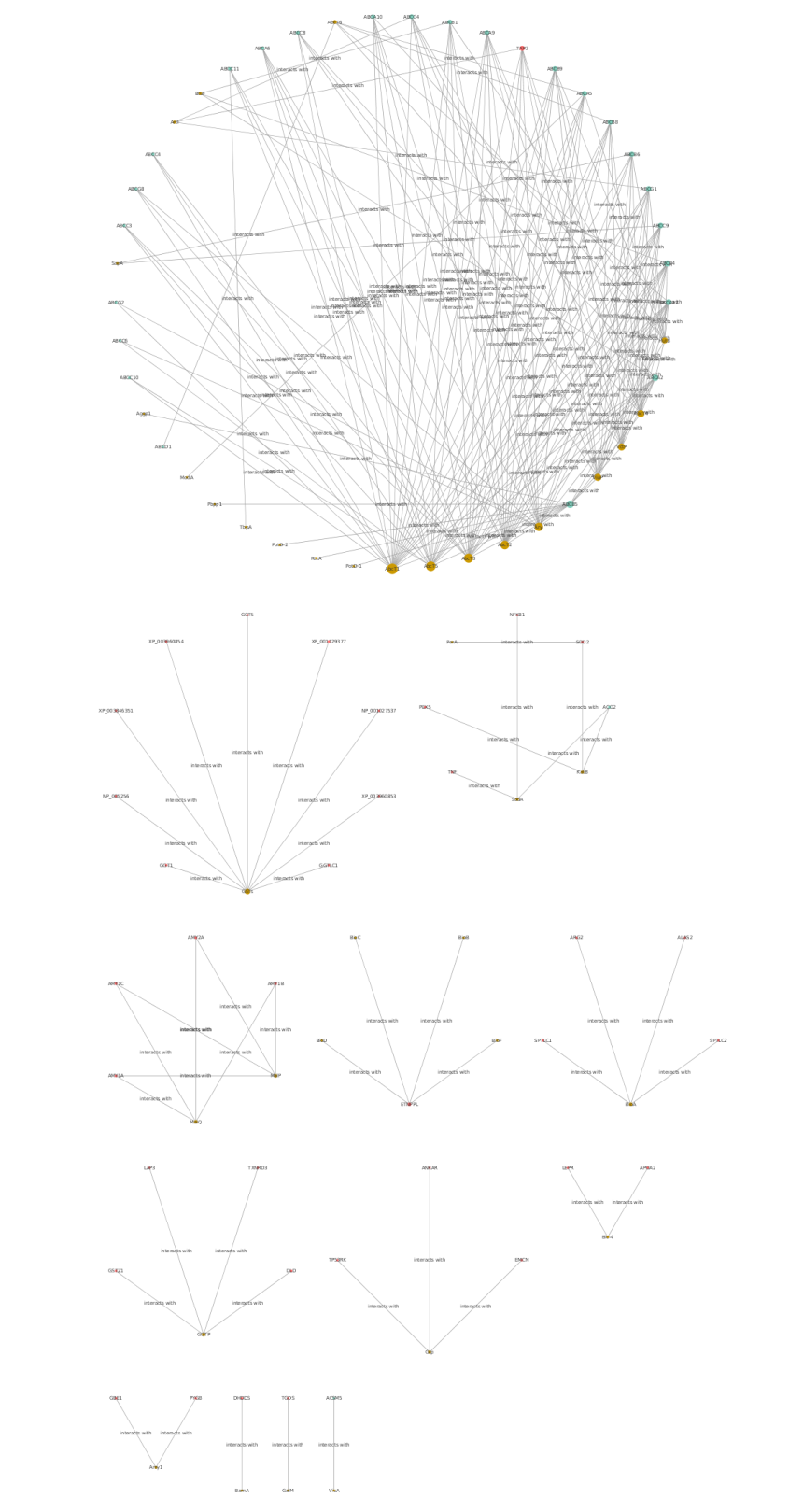


**Figure S4:** Probable role of bacterial TesA in the formation of lipid rafts during *V.cholerae* infection.


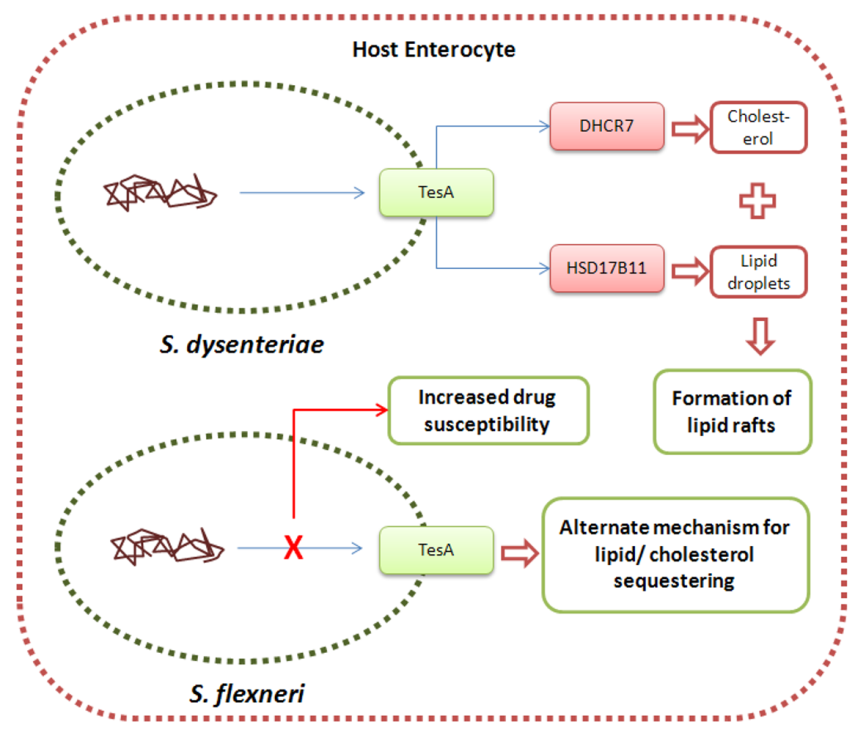


**Appendix 1**

**List of human proteins that were closely associated with the human–*Escherichia coli* PPI network.**

MD-2; TRL5; TRL4; CD14; TUBA; TUBB; GEF-H1; Rho; ROCK; ITGB1; c-ABL1; Nck; N-WASP; Cortactin; F-actin; Cdc42; CK-18; PRKCA; Ezrin; CLDN1; Cadherin; beta-Catenin

**List of human proteins that were closely associated with the human–*Salmonella enterica* PPI network.**

LBP; TLR4; CD14; ZO-1; Myd88; IPAF; ASC; CASP1; Rac1; Cdc42; RhoG; p38; ERK1/2; JNK; AP1; NF-kappB; WAVE; N-WASP; Apr2/3; F-actin; PKN-1; IL-18; IL-1beta; IL-1alpha; IL-6; IL-8; MIP-1alpha; MIP-1beta; MIP-2KC; GM-CSF; ROCK; Myocin II; Kinesin; Rab7; RILP; Dynein; Filamin; INF-gamma; INFGR1; INFGR2; iNOS

**List of human proteins that were closely associated with the human–*Shigella* *dysenteriae* PPI network.**

Integrin-alpha; Integrin-beta; CD44; c-Src; Cortactin; Ab1; Crk; Cdc42; Vinculin; mDia1; Rock; ELMO; Dock180; Rac1; WAVE; F-actin; N-WASP; Atg5; Mad2L2; Nod1/2; RICK; IKK-alpha; IKK-beta; IKK-gamma; UbcH5b; beta-TrCP; JNK; ERK1/2; p38; NF-kappaB; I-kappaB; U2AF^35^; IL-8

**List of human proteins that were closely associated with the human– *Shigella flexneri* PPI network**.

Integrin-alpha; Integrin-beta; CD44; c-Src; Cortactin; Ab1; Crk; Cdc42; Vinculin; mDia1; Rock; ELMO; Dock180; Rac1; WAVE; Arp2/3; F-actin; N-WASP; Atg5; Mad2L2; Nod1/2; RICK; IKK-alpha; IKK-beta; IKK-gamma; UbcH5b; beta-TrCP; JNK; ERK1/2; p38; NF-kappaB; I-kappaB; U2AF^35^; IL-8

**List of human proteins that were closely associated with the core HPI network.**

TLR4; CD14; Rho; ITGB1

**Appendix 2**
